# Events and Causal Mappings Modeled in Conceptual Spaces

**DOI:** 10.3389/fpsyg.2020.00630

**Published:** 2020-04-07

**Authors:** Peter Gärdenfors

**Affiliations:** ^1^Department of Philosophy and Cognitive Science, Lund University, Lund, Sweden; ^2^Palaeo-Research Institute, Faculty of Humanities, University of Johannesburg, Johannesburg, South Africa

**Keywords:** causation, robotics, events, action, conceptual space

## Abstract

The aim of the article is to present a model of causal relations that is based on what is known about human causal reasoning and that forms guidelines for implementations in robots. I argue for two theses concerning human cognition. The first is that human causal cognition, in contrast to that of other animals, is based on the understanding of the *forces* that are involved. The second thesis is that humans think about causality in terms of *events*. I present a two-vector model of events, developed by Gärdenfors and Warglien, which states that an event is represented in terms of two main components – the force of an *action* that drives the event, and the *result* of its application. Apart from the causal mapping, the event model contains representations of a patient, an agent, and possibly some other roles. Agents and patients are objects (animate or inanimate) that have different properties. Following my theory of conceptual spaces, they can be described as vectors of property values. At least two spaces are needed to describe an event, an action space and a result space. The result of an event is modeled as a vector representing the change of properties of the patient before and after the event. In robotics the focus has been on describing results. The proposed model also includes the causal part of events, typically described as an action. A central part of an event category is the mapping from actions to results. This mapping contains the central information about *causal* relations. In applications of the two-vector model, the central problem is how the event mapping can be learned in a way that is amenable to implementations in robots. Three processes are central for event cognition: causal thinking, control of action and learning by generalization. Although it is not yet clear which is the best way to model how the mappings can be learned, they should be constrained by three corresponding mathematical properties: monotonicity (related to qualitative causal thinking); continuity (plays a key role in activities of action control); and convexity (facilitates generalization and the categorization of events). I argue that Bayesian models are not suitable for these purposes, but some more geometrically oriented approach to event mappings should be used.

## Introduction

Causal reasoning is a central cognitive competency, allowing us to reliably, albeit not perfectly, predict the future and to understand the causes of events that we observe. This form of reasoning has been studied extensively in psychology and philosophy (see e.g., Waldmann and Hagmayer (2013) for an overview). In this article, my focus will be on aspects of human causal reasoning that should be considered when developing robotic systems that are capable of similar forms of reasoning.

If we want to develop efficient systems for human-robot interaction, the best way is to have robots reason about causes in the same way as humans do. Therefore, we need a model of human causal cognition that allows implementation. Pearl (2018) writes that we recognize human reasoning “through words such as ‘preventing,’ ‘cause,’ ‘attributed to,’ ‘discrimination,’ and ‘should I.’ Such words are common in everyday language, and our society constantly demands answers to such questions. Yet, until very recently science gave us no means even to articulate them, let alone answer them. Unlike the rules of geometry, mechanics, optics or probabilities, the rules of cause and effect have been denied the benefits of mathematical analysis.”

This article will argue for two theses concerning human cognition. The first is that causal cognition is based on the understanding of the *forces* that are involved. In the section Causal Reasoning with Forces, I present some data concerning the differences between human causal reasoning and that of other animals. I propose that the best way to understand these differences is that humans have evolved mental representations of the forces behind an action or a physical process that lead to an effect.

The second thesis is that humans think about causality in terms of *events*^1^. However, unlike other models in philosophy and psychology where causality is seen as a relation between events, the model presented here moves causality inside events in the sense that an event is modeled as containing two vectors representing a cause as well as a result. In Section 3, I present a model that is based on a mapping from actions to results. The purpose of such a mapping is to represent causal relations. Actions are modeled in terms of forces, while effects are modelled as different kinds of changes, for example, a change in the physical location or a change of some property of the agent. Apart from the causal mapping, the event model contains representations of an agent, a patient and possibly some other roles.

Three cognitive processes crucially depend on event cognition: causal thinking, control of action and learning by generalization. All three processes are important for robot applications. The central problem is to model the event mapping and how it is learned in a way that is amenable to implementation.

The mapping from forces to results may have a complicated structure due to context dependent or unknown counterforces. However, the mapping is constrained by three properties that correspond to the three cognitive processes respectively: (1) Larger forces lead to larger results (related to qualitative causal thinking); (2) small changes in the force lead to small changes in the result (plays a key role in action control); and (3) intermediate results are caused by intermediate forces (facilitates generalization and the categorization of events). These properties will be presented and analyzed in the section Three Constraints on the Causal Mapping.

On the basis of the event model and the constraints on the causal mapping, I will discuss some ideas about how such mappings can be handled in a robot. This will be the topic of in the section Implementing the Event Model and the Causal Mapping in Robots. The main problem to be solved is how the event mapping from causes to effects can be learned. Here the three constraints turn out to be central. I also argue that Bayesian models are not appropriate since they cannot account for the three constraints on the causal mapping in a natural way.

## Causal Reasoning With Forces

### Human Reasoning About Forces

The sensory influx to the human brain is extremely rich – a “blooming buzzing confusion” according to James (1890, p. 42). It is something of a wonder that the brain can sort up the information received by our senses. In particular, it has a capacity to discover causal relations between complex phenomena. It is, however, still largely an open question how this mechanism works.

There are several proposals for how to analyze causal cognition. Gärdenfors (2003, Section 2.8) distinguishes between four kinds of causal reasoning: (a) Being able to foresee the physical effects of one’s own actions (the first type to develop in infants); (b) being able to foresee the effects of others’ actions; (c) understanding the causes of others’ actions; and (d) understanding the causes of physical events. Along similar lines, Woodward (2011) distinguishes between *egocentric learning*, which is the ability to learn that one’s own physical actions can cause certain outcomes. The second kind is *agent causal learning*, when one also learns about cause from the actions of others. The third kind is *observation/action causal learning*, when one is able to integrate a natural signs or patterns with the other two types of learning^2^.

The models indicate that being able to categorize actions is a necessary prerequisite for understanding causal relations. Psychological studies have established that the brain processes lead to a considerable information reduction when actions are classified. For example, Johansson (1973) showed that the kinematics of a movement contain is sufficient to categorize an action. He attached light bulbs to the joints of actors who were dressed in black and moved against a black background. The actors were then filmed while performing bodily actions such as walking, running and dancing. When subjects saw the movies, in which only the dots of light could be perceived, they correctly categorized the actions within a few hundred milliseconds.

The upshot of these experiments is that the kinematics of a movement contains information that is sufficient for the identification of the underlying dynamic force patterns, that is velocities and accelerations (Runesson, 1994). Further psychological evidence [Wolff (2007, 2008), Wolff and Shepard (2013), Wolff and Thorstad (2017)] supports that people can directly perceive the *forces* that control different kinds of motion. In other words, the sensory input generated by the movements of an individual (or an object) is sufficient for the brain to calculate the forces that lead to the movements. The process is automatic: people cannot help but seeing the forces.

In the philosophical literature, a cause has mainly been viewed as something that makes a difference with respect to some effect. The differences are typically analyzed in terms of co-variations (see Waldmann and Hagmayer, 2013 for a presentation). However, nothing is said about *how* it makes a difference. Theories of causation that are based on forces provide an explanation (Wolff, 2007). Forces also open up for new empirical methods to study casual relations that go beyond covariations.

The capacity to understand the role of physical forces, not just forces involved in animal actions, develops early in human infants. Michotte (1963) showed that if one object moving on a screen collided with another object and the other object started moving in the same direction, then adults perceived the launching of the second object as caused by the movement of the first. In contrast, if the second object only started moving half a second after the collision, then the delay destroyed the impression of causality. Leslie and Keeble (1987) performed Michotte’s experiments with six-month-old infants and showed that they reacted differently to the two types of events. Leslie (1995) concludes that infants have a special system in their brains for mapping the ‘forces’ of objects.

### Animal Reasoning About Forces

It seems that non-human primate reasoning about forces is less developed compared to that of humans. For example, in his early experiments on chimpanzee planning, Köhler (1917) observed that apes had great difficulties in stacking boxes on top of each other. He notes about Sultan, the best problem solver among the chimpanzees, that when he tried to put a second box on top of a first, “instead of placing it on top of the first, as might seem obvious, began to gesticulate with it, … he put it beside the first, then in the air diagonally above, and so forth.” After similar observations on other apes, Köhler (1917, p. 149) concludes that “there is practically no statics to be noted in the chimpanzee.” For more experiments in the same direction see Tomonaga et al. (2007) and Cacchione et al. (2009). These observations indicate that apes in general do not have a well-developed understanding of the role of gravitation on other objects than their own bodies.

Povinelli (2000) also performed a series of experiments indicating that chimpanzees and other primates are very limited in their capacities to reason about gravitation. These experiments have been followed by a series of others (e.g., Call, 2010; Hanus and Call, 2008; Martin-Ordas et al., 2008; Penn and Povinelli, 2007), and they have generated an extended debate (see Seed and Call, 2009; Seed et al., 2011). Povinelli and Penn (2011, p. 77) conclude that “only humans are capable of second-order relational reasoning, and only humans, therefore, have the cognitive machinery that can support higher-order, theory-like, causal relations.” In line with this, Johnson-Frey (2003: 201) writes: “Comparative studies of chimpanzee tool use indicate that critical differences are likely to be found in mechanisms involved in causal reasoning rather than those implementing sensorimotor transformations.”

Furthermore, in a comparative study of on nut-cracking in humans and chimpanzees (Boesch et al., 2017), it was found that humans understood how to apply force to extract numerous nut species through using hammerstones. Yet, the chimpanzees only ever applied such force to Panda nuts, even though they regularly eat hard Irvingia nuts using their teeth. This is a good example of how humans, compared to chimpanzees, have a more abstract causal understanding of tool-assisted force application, allowing us to apply similar solutions to a wider range of subsistence problems. By adding the ability to mentally represent detached forces – and not just actions – as causes, the human mind evolved to extend its capacities to reason and to plan beyond that of other primate species. Gärdenfors and Lombard (submitted) argue that this development was driven (at least in part) by more advanced tool use and manufacturing.

### A Cognitive Approach to Causation

This comparison between the causal reasoning of humans and other animals provides a reason for focusing on models that are based on forces also in developing causal reasoning in robots. In the following section I present a model that can function as a framework for computational implementations.

The basic ontological position of my approach to causal reasoning is that causes are cognitive constructions and not relations in the real world. In other words, my account is cognitivist rather than realist. For an argument for this position see Wolff (2007, p 7).

Another central aspect is that the forces of an agent are not the only elements involved in human causal judgements, but counterforces of various kinds (forces exerted by a patient or contextual forces such as gravitation) are also taken into account. This aspect is included in Talmy’s (1988) ‘force dynamics’ and is further developed in Wolff’s (2007, 2008, 2012) ‘dynamics model’. Wolff (2007) has shown that adults can combine different kinds of forces in their reasoning. For example, they can estimate the combined forces of a boat motor and the wind and their effects on how the boat crosses a lake. Depending on how the ‘affector’ force vector (produced by an agent) combines with a ‘patient’ force vector to generate a ‘result’ vector, subjects judge that the affector force either *causes*, *enables* or *prevents* an effect. These results indicate that subjects cognitively distinguish between different kinds of causal relations. Talmy’s force dynamics is grounded in physical events, but it is also used to understand psychological or social interactions.

Göksun et al. (2013) extended Wolff’s experiments to a study of 3- to 5-year-olds who, in addition to one-force events, were asked to predict the path of a ball that was influenced by two forces that were combined to represent force dynamics patterns of ‘cause’, ‘enable’ and ‘prevent’. The study showed that while the children were successful in their causal reasoning about the one-force events, they attended less to a second force, incorporating it only in the case both forces acted in the same direction. The older they were, the more successful the children became in reasoning about the effects of the second force (George et al., 2019). These experiments indicate that human abstraction and reasoning about physical forces develop with experience over age, even though the general system for perceiving forces as causes is present already at an early age.

## A Cognitive Model of Events

### A Two-Vector Model of Events

The second thesis of this paper is that human causal cognition is structured in terms of *events*. This section argues that mental representations of events exploited in language, physical thinking and planning can be modeled in geometric terms. Several authors (e.g., Talmy, 1988; Croft, 2012; Wolff, 2007, 2008, 2012; Gärdenfors and Warglien, 2012; Gärdenfors et al., 2018) have adopted such a geometric perspective on events. Following earlier work on conceptual spaces (Gärdenfors, 2000, 2014, Gärdenfors and Warglien, 2012; Warglien et al., 2012), I model events as complex structures that involve an action space based on forces and other spaces representing the results of actions.

The two-vector model states that an event is represented in terms of two components – the force of an *action* that generates the event, and the *result* of its application. Both components are represented as vectors in spaces. (In the special case when there is no change, that is, when the result vector is the zero vector, the event is a *state*.) The result of an event is modelled as a vector representing the change of properties of the patient before and after the event.

As a simple example of the model, consider the event of Oscar pushing a table. The force vector is generated by the agent Oscar. The result vector is a change in the location of the patient – the table – and thus a change in the properties of the table. The exact result vector depends on the properties of the table, for example its weight as well as other forces in the context, for example, friction. Although typical event representations contain an agent, some need not involve any: for example, events of falling, drowning, dying, growing and raining. The force and result vectors are central, but more vectors and objects may be involved in representations of events as I show below. Following Gärdenfors and Warglien (2012), I put forward the following requirement on the cognitive representation of an event:

*The two-vector condition*: An event must contain at least two vectors and one object; these vectors are a result vector representing a change in properties of the object and a force vector that causes the change.

The central object of an event will be called the *patient*. If there is an entity generating the force vector, it will be called the *agent* (Wolff, 2007) calls them *force recipient* and *force generator*, respectively). Agents and patients are objects (animate or inanimate) that have different properties. Following my theory of conceptual spaces (Gärdenfors, 2000, 2014), they can be described as vectors of values from property dimensions.

At least two spaces are needed to describe an event, an action space and a result space. The action space can be conceived as a space of forces (or, more generally, force patterns) acting upon some patient, the properties of which are described in the result space. The spaces represent different types of vectors: forces have a different nature than changes in properties.

As the result component of the event represents changes in the properties of the patient, the result space can also be modeled as a vector space. The result vectors typically stand for changes of location or changes of object properties. For example, when Lucy opens the door, the agent Lucy exerts a force vector (action) on the door that leads to a change of the position of the door (result). Or in the event of the storm felling a tree, the force of the wind (action) leads to a change of the direction of the tree (result).

Events are represented not only as single instances, but more generally as event *categories*, for example, throwing a ball. The description of change vectors can be generalized to that of change *vector fields* by associating to each action force vector a result vector, taking into account the (counter-)forces exerted by the patient and other contextual forces. Mathematically, such a mapping from actions to results can be seen as a function from a force vector that is the resulting combination of the action vector and other contextually given forces to a result vector (see Gärdenfors and Warglien (2012) for a more detailed description of the mapping). This mapping is part of the representation of an event category and it contains the central information about causal relations.

The events need not only involve physical forces, but also mental ‘forces’ can be causal variables (Talmy, 1988; Leslie, 1994). Humans interpret many mental factors (for example commands, threats, insults and persuasive arguments) as forces that can create a change in the physical, cognitive or emotional state of the addressee. For example, Wolff (2007, pp. 19– 22) presents two experiments where a woman intends to cross a street to meet (or to avoid) a man and the directions of a police man in the street crossing acts as an additional ‘force’ that enables or prevents the woman from reaching her goal. The results show that the subjects interpret the woman’s intention as a force and they describe the various scenarios in the same terms as they would use for a situation where only physical forces are involved. In other examples, such as a case of threatening, the resulting change is not physical, but it can still be represented in terms of changes in a conceptual space (assuming that the concept ‘person’ has a space of emotional states). Wolpert et al. (2003) present an analysis of how this kind of reasoning can be modeled in terms of control theory.

The forces can also be medical, economic or social (Talmy, 1988). For example, in “The aspirin caused his headache to go away,” the medicine acts as ca force causing a change in his physical state. And in “The high price offered enabled her to sell her mother’s wedding ring,” the price acts as a force. A social example is “The pressure from the villagers caused him to mow his lawn, even though he wanted to keep it as a meadow.”

I next turn to a more detailed description of the two main components of the model.

### Representing Actions

Following Gärdenfors (2007a) [see also Warglien et al. (2012) and Gärdenfors (2014)], I proposed in the previous section that the human cognitive processes extract the forces that generate different kinds of actions. This leads me to the following thesis:

*Representation of actions*: An action is represented by the pattern of forces that generates it.

The thesis speaks of a pattern of forces since, for most bodily actions, more than one body part is moving. Therefore, multiple force vectors are acting in parallel [this is analogous to Marr and Vaina’s (1982) differential equations]. The patterns of forces can be described in the same way as the modeling of shapes in Gärdenfors (2014, Section 6.3). Like shapes, force patterns also exhibit meronomic relations. For example, a bird with short wings flies in a different way than a bird with a large wing span.

In order to investigate the action space, judgements of similarities between actions can be used. The methods for estimating similarities between objects are essentially the same as for objects. The dynamic properties of actions are in focus for such judgments: for example, throwing is more similar to waving than to crawling. A large set of such similarity ratings can serve as data for one of several related statistical techniques, such as multidimensional scaling or principal component analysis that turn similarities into spatial structures. The geometric structure of the action space is largely unknown, except for a few recent studies that are presented below. In line with other domains, it is assumed that the notion of betweenness is meaningful in the action space. This allows me to formulate the following thesis [which is parallel to the thesis about properties in Gärdenfors (2000, 2014)]:

*Thesis about action concepts*: An action concept is represented as a convex region in the action space.

It is natural to interpret convexity as the assumption that, for any two actions that fall under an action concept, any linear morph between the actions will also belong to the same concept.

Empirical support for the thesis about action concepts involving body movements is presented by Giese and Lappe (2002). Starting from Johansson’s (1973) patch-light methods, they edited videos of bodily actions such as walking, running, limping, and marching. Linear combinations of the positions of the joints of the body were created and they then created videos exhibiting morphs of the recorded actions. Subjects who watched the morphed videos were asked to categorize the actions. Giese and Lappe did not explicitly investigate whether the action categories that the subjects created correspond to convex regions. The data they present clearly support convexity.

Another example is Slobin et al. (2014), who investigated how subjects categorized actions shown in 34 video clips of motion events such as walking, running and jumping, The subjects, who were native speakers of English, Polish, Spanish, and Basque, were asked to put a label, as precise as possible, on the action they saw in the clips. Based on the answers a two-dimensional multidimensional scaling solution was calculated. The result indicates that four separated convex regions emerge for each of the languages studied. These regions correspond to walking, running, crawling, and to some non-canonical actions (such as leaping or galloping). Together with similar results from Malt et al. (2014), these results provide support for the thesis about action concepts. However, for human-robot applications, more research concerning the structure of action space is required.

In robotics, the work has mainly dealt with how the *results* of actions can be modelled [e.g., Cangelosi et al. (2008), Lallee et al. (2010), and Demiris and Khadhouri (2006)]. In human-robot interaction, however, it is more important that the robot can categorize human and other actions by the *manner* they are performed. This is called recognition of biological motion (Hemeren, 2008; Gharaee et al., 2017a, b). Categorizing actions is particularly important if the goal of the robot is to understand the intentions behind the actions.

### The Causal Mapping

The main reason for introducing the event model is that it is a natural way of capturing how we think about causation: the action *causes* the result. In the literature, most authors analyze the causal relation between the action and the effect as holding between two events (see e.g., Zacks and Tversky, 2001; Casati and Varzi, 2008). In contrast, the model presented here describes causation as a relation *within* an event. Furthermore, the distinction between forces and changes of states also means that the cause and the result, in contrast to traditional theories, are modelled as two different entities.

There are many similarities between the event model presented here and Wolff’s (2007, 2008, 2012) dynamics model. His affector vector corresponds to the force vector, his patient vector to the counterforces, and he also includes a result vector. The two models have been developed for slightly different purposes: the two-vector model is presented as a general model of events while the focus of Wolff’s model is on causal reasoning. Another difference is that his result vector is of the same kind as the force vectors. In contrast, in the model presented here causes and effects modeled as entities of the different types: they belong to different spaces – causes to the force space and results to change in location space (in the case of movements) or in some property space (color, size, shape, weight, temperature, etc.).

The two-vector model of events has testable consequences. Wolff (2007) presents a study which shows that individuals can perform intuitive addition of force vectors when observing two force simultaneously affect the trajectory of a patient). Michotte’s (1963) ‘launching’ experiments show that how subjects attribute causality in a simulated event involving an object A that hits an object B A depends on the angle of the trajectories of A and B. This shows that subjects judge whether an animation represents one or two events depending on how forces are mapped onto movements. The perception of such a mapping has been shown to be remarkably precise, and to predict the ‘causal impression’ on the subjects (White, 2012). In these cases, the two-vector model of events predicts well how individuals perceive causal events.

The event model, can handle *what-if* questions, that is, counterfactual reasoning concerning what would have happened if an action would have been different. For example: “If I had dropped the glass on the ceramic floor instead of on the mat, then it would have broken.” Such reasoning can be computationally modeled by simulations of various changes in the force and counterforce vectors and using the mapping function and assumed counterforces to predict a result. Simulations use similarity measures and operations for projecting forwards and backwards to understand the causes and consequences. For example, Johnston’s (2009) COMIRIT system can be used to integrate commonsense reasoning and the geometric inference of conceptual spaces. COMIRIT establishes a mechanism for assigning ‘semantic attachments’ to symbols in knowledge representations systems that can be used to automatically construct simulations and utilize machine learning methods. In contrast, probabilistic models of causation, which will be discussed in the subsection Why Probabilistic Models Are Not Suitable, have deep-going problems in handling what-if reasoning (Pearl, 2018).

Similar to counterfactual reasoning, humans often reason in terms of *omissive* causation, that concerns events that do not occur. For example, the fact that a person did not fill in his tax forms, caused that he was fined by the tax authorities. This is a problem for many other models of causation, but the two-vector model can also explain omissive causation [for related solutions see Talmy (1988); Wolff et al. (2010), and Wolff and Thorstad (2017)]. To illustrate how the two-vector model applies in such cases, consider the famous gag in the movie *A Night in Casablanca* where Harpo Marx is leaning against the wall of a house. A policeman comes up to him and says “What do you think you are doing? Holding up the building?” Harpo nods energetically with his typical smile but the policeman chases him away. In the background one sees how the building crashes into the ground. Here, the crash is caused by Harpo’s omission of supporting the wall. In the terms of the two-vector model, the force vector from Harpo towards the wall generates a stable state where the wall is in balance despite its counterforces. When Harpo’s supporting force is eliminated, the counterforces generate the crash of the house.

## Three Constraints on the Causal Mapping

Given our ignorance of the counterforces in a situation and the limited knowledge about the relevant causal relations, it is often very hard to precisely predict the outcome of an action. Still, the qualitative effect of actions can be understood.

When it comes to computational implementations of the two-vector model in a robotic system, the mapping between the force space and the result space is the most central part of the event model. A problem is that externalities, such as friction and other counterforces, make it difficult to determine the result vector, given the force vector. For example, pushing a coffin may result in the coffin moving, other times not; taking a medicine sometimes cures a patient, other times not.

The formal nature of event mappings has been little investigated. Although other theories of events (Talmy, 1988; Croft, 2012, Wolff, 2007, 2008) also build on such a mapping, they do not analyze it. Gärdenfors et al. (2018), however, present an analysis of three general principles for event mappings, that constrain the relation between the force vector and the result vector. All three principles are of a qualitative form, which reflects the qualitative nature of event cognition. They function as ceteris paribus constraints.

As a background for the principles, note that there are three central cognitive processes that depend on mental representations of events: causal thinking, control of action, and learning. These are characterized respectively by three qualitative properties that are central for the corresponding processes: (1) larger forces lead to larger results (this relates to qualitative causal thinking); (2) small changes in forces lead to small changes of the result (this is important for action control); and (3) intermediary results are caused by intermediary forces (this facilitates generalization and categorization of events). Mathematically, these properties correspond respectively to the monotonicity, continuity and convexity preservation of the mapping from actions to results. The motivation for investigating them is that human causal thinking typically satisfies these properties. The three properties thus impose constraints on the mapping from actions to events, something which is crucial when such a mapping is to be learned by a robot.

### Larger Forces Lead to Larger Results

A general constraint for qualitative causal thinking is that whenever counterforces and other external factors are kept constant in a given situation, then increasing the force involved in the action will also lead to a larger result (or at least not decrease it). For example, if I push the gas pedal harder in my car, it will run faster.

This constraint captures an important part of our reasoning about how a change of an outcome depends on a change of an action. The constraint makes possible qualitative predictions about the effects of actions. It is a central component in interpreting causality (Hume, 1748/2000; Wolff, 2007, 2008) and in making causal inferences.

The constraint enables qualitative causal inferences. First of all, it makes it possible to draw basic inferences about how changes in causes will lead to changes in effects. For example, since different individuals may react with different intensity to a medicine, it is difficult to predict the size of the effect. One may, however, still make the prediction that increasing the dose of the medicine will increase the effects. Mill (1843) dubbed this form of inference ‘the method of concomitant variations’.

Mathematically, this constraint corresponds to the *monotonicity* of the mapping function. A function is said to be monotonous when f(x) ≤ f(y), whenever x ≤ y. This property thus depends on an ordering relation on the forces. As long as all forces act in the same direction such an ordering exist. However, in higher dimensional spaces such an ordering function may not exist.

The constraint that larger forces lead to larger results can also support reverse inference processes. When wanting to identify the relevant causal factors among multiple potential ones, the constraint can provide a powerful selection criterion. For example, the tides have been observed as long as humans have existed, but it was only when the correlations to the moon’s position and distance was discovered, taken together with Newton’s law of gravitation, that we understood the force vectors causing the tides.

### Small Changes in the Force Lead to Small Changes in the Result

When the aim is to change the effect of an action only by a small amount, it can be achieved by applying a correspondingly small change of force. For example, when turning the control for a heater on a stove a little more to the right, one expects the heat also to increase just a little, and not lead to a drastic change that would destroy the food. And when a tennis ball is hit a little harder, it will fly a little faster and further, but not move wide out of the court.

Mathematically, this constraint corresponds to the *continuity* of the mapping function. This can be defined in terms of a nearness relation on the space, which is easily defined for the force space^3^.

Central both to human and robotic actions is *motor control*, which in general requires the fine-tuning of an agent’s forces (Wolpert and Flanagan, 2001; Stolt et al., 2012). For example, balancing a stick on a finger requires very small adjustments in the neighborhood of the equilibrium position (see e.g., Shiriaev et al., 2007).

While the constraint captures a very general principle of causal thinking, it is not always true that small changes in the force lead to small changes in the result. Sometimes small changes lead to phase transitions. For example, if you are gradually increasing your arm force when bending a wooden stick, there is a point where the stick breaks. At the transition point, a very small change of effort produces a large effect. In more general terms, a discontinuous phase transition occurs when an obstructing counterforce is suddenly overcome, and a drastically different result is achieved.

### Intermediate Results Are Caused by Intermediate Forces

Imagine that you are throwing a ball at a basket. You can control the forces of your arms in the throw. If you have tried force *x* and observed that the ball was short of the basket and tried force *y* and observed that the ball went too far, then you presume that a force of a strength between *x* and *y* will lead to an intermediary result.

The third constraint can be formulated as that the causal mapping *f* is convexity preserving: if the force vector *z* is between force vectors *x* and *y*, then the result *f(z)* is between the results *f(x)* and *f(y).* In other *words, intermediate forces lead to intermediate results.* Therefore, this constraint depends on the fact that betweenness is defined for the force and result spaces^4^.

This constraint applies to many situations involving bodily movement. A clear example comes from Runesson and Frykholm (1981) who showed subjects patch light movies of a person lifting objects that weighed between two and twenty kilos. The objects themselves were not visible in the movies but only the movement patterns of the person lifting them. In spite of this limited information, the subjects could very accurately predict the weights of the object. The upshot is that the movement patterns were sufficient for the subject to infer the forces that the person lifting the box was applying. The subjects then inferred that intermediary forces corresponded to intermediate weights of the boxes. I am not claiming that the inference is conscious, only that our causal reasoning obeys the constraint.

I have argued that the process of *learning* new concepts requires regions that represent concepts to be convex in order for the process to be efficient (see Gärdenfors, 2000, Ch. 3 and Gärdenfors, 2001). Furthermore, convexity also makes *generalization* efficient since, by interpolation, inferences over whole regions can be made given only a limited number of observations. Finally, feedback control mechanisms also require that the mapping from actions to results preserves convexity (e.g., Shiriaev et al., 2007).

It should be noted that generalization in psychology has focused on generalizing from a particular data point (for example Shepard, 1987). However, generalizing by interpolation between data points is at least as important. Given that convexity is satisfied, it is sufficient to know the mappings from two force vectors to two result vectors to know what lies between them. Thus, convexity helps to predict unspecified properties of the event^5^.

To sum up this section, the three qualitative constraints do not uniquely determine the mapping from causes to results, but they add rich structure to it. The constraints make it possible to draw robust inferences even if counter-forces and other contextual factors are unknown. In this way, the constraints considerably strengthen human causal thinking. It is therefore recommendable that robotic systems for causal reasoning also obey these constrains.

The three constraints have been presented here as part of the two-vector event model presented in Section 3. Because of their general nature, however, they can also be applied to other models such as the force dynamics of Talmy (1988), the dynamic model of Wolff (2007, 2008) and the event representations in Croft (2012).

## Implementing the Event Model and the Causal Mapping in Robots

The core of the two-vector model of events consists of the mapping between the force space and the result space. In this section, I present some considerations on how the mapping – and how it is learned – may be implemented in a robotic system.

### Learning the Event Mapping: Computational Aspects

As a simple but illustrative case, I will take Wolff’s (2007, 2008) studies of how people evaluate causes and effects of how controlling the speed and direction of the motor of a boat will affect its trajectory. A complicating factor is that, apart from the resistance of the water, there is an unpredictable wind that acts as a counterforce. In this causal web, the physics of the situation allows a system to learn the unknown variables. Firstly, in situations without wind the effects of the speed and direction of the force vector of the motor can be learned (and it will be a linear mapping as long as friction is constant), since the friction vector is always in the opposite direction of the force vector. Secondly, once this mapping is learned, one can simulate situations where there is a wind, and by adding the friction and wind counterforce vectors, the system can learn to identify a motor force vector that will result in the desired effect. There are several ways of computationally implementing such a learning system by using traditional physical modeling or by using some form of neural network. I will not go into details here.

Other situations will not admit such a principled learning procedure. In many cases there may be unknown counterforces and other factors that make the mapping non-linear and dependent on several external variables. However, by letting the system experience a number of varied data points, approximations of a mapping function can be calculated. When a so far unobserved result vector is desired, interpolations of force vectors resulting in similar effects can be used to generate a new force vector that, because of the three constraints of the mapping function, result in an approximate result vector. For the implementation of learning situations of this kind, many methods from control theory can be employed (see e.g., Ardakani et al., 2019).

Even in situations where the forces are non-physical, similar methods can be used to learn the event mapping. For example, Wolpert et al. (2003) explore the computational parallels between motor control, on the one hand, and action observation, imitation, and social interaction, on the other (see also Gärdenfors, 2007b). They argue that motor commands that generate bodily actions can be extended to social actions directed towards other people. In this extension, the changes in the state of my body correspond to changes of the state of mind of another person.

Another field of learning that is required for robotic reasoning about causation and for communicating, for example in a planning situation, is *action categorization*. Representations of actions in terms of conceptual spaces, such as those proposed by, for example, Chella et al. (2001), Gärdenfors (2014), and Gharaee et al. (2017a, b), provide a potentially fruitful method for implementations. Simulating an action and then using the event mapping that has been learned to predict a result vector, can then be used to generate plans and to reason about complex situations. In this way, simulations can provide the robotic system the power to imagine events that is needed to understand the physical, social and, eventually, the emotional world we live in.

The event structure has not yet been implemented in any concrete system. However, a cognitively motivated architecture for holistic AI systems, including robotic ones, that integrates machine learning and knowledge representation has been proposed in Gärdenfors et al. (2019). The central idea of the proposal is to use ‘event boards’ representing components of events as an analogy to blackboards that formed the backbone in some earlier AI systems. The event components that are placed on the board are represented by vectors in conceptual spaces rather than in symbolic structures that has been used in previous systems. A control level that is added to the event board includes an attention mechanism that decides which processes are run.

### Why Probabilistic Models Are Not Suitable

Within computer science, Bayesian models or Bayesian nets are popular statistical tools since they require minimal prior knowledge (see Waldmann and Hagmayer, 2013 for a presentation). For example, ‘constraint-based algorithms’ allow the derivation of causal structures on the basis of the pattern of statistical dependencies of a set of variables (see e.g., Pearl, 2000). Another way of learning causal structure is to formulate the problem in terms of Bayesian inferences. For such a learning mechanism, the learning system (for example, a robot) must determine the probability of a causal structure given the available data. There also exist proposals for hybrid systems combining Bayesian models with more traditional models (Waldmann and Mayrhofer, 2016).

There are, however, some problems connected with probabilistic models (Wolff, 2007; Waldmann and Hagmayer, 2013), in particular when it comes to implementations on robotic systems. In experimental studies, subjects have had difficulties in extracting causal relations based on covariation data even though these experiments typically present a small number of variables (Steyvers et al., 2003). For humans, a single instance of a causal connection is sufficient to pick up a causal relation and it would be desirable that a robotic system has a similar capacity. Such a rapid process is difficult to capture in a probabilistic model. According to the model presented here, the forces that generate an action are essential for causal inferences and such forces are, in general, inaccessible to probabilistic approaches. In brief, Bayesian processes are computationally not suitable for implementations in robotic systems.

The implausibility of domain-general algorithms of structure induction has led Waldmann (1996) to propose the view that people generally use prior hypothetical knowledge about the structure of causal models to guide learning in a top down fashion, so called knowledge-based causal induction. In line with this, also Waldmann and Hagmayer (2013) argue that causal cognition of people cannot be encompassed by the Bayesian formalism. For these reasons, I do not consider the Bayesian approach to be a viable alternative for robotic systems^6^. Furthermore, the use of the general principles of monotonicity, continuity and convexity makes much of Bayesian reasoning unnecessary.

## Conclusion

In this article, I have argued for two theses. The first thesis is that human causal cognition (in contrast to that of non-human animals) build on understanding the *forces* that are involved in an action that leads to a result. The second thesis is that humans think about causality in terms of *events*. I have presented the two-vector model of events that is based on conceptual spaces and shown that it captures several aspects of human causal reasoning.

I have argued that Bayesian models are not suitable for representing causal structures, in particular not the event structures that have been presented here. The two-vector model of events generate new types of problems that must be solved in order to create robotic systems capable of causal reasoning. The main problem is to devise methods for learning appropriate mappings from actions to results, that is, from causes to effects.

## Author Contributions

The author confirms being the sole contributor of this work and has approved it for publication.

## Conflict of Interest

The author declares that the research was conducted in the absence of any commercial or financial relationships that could be construed as a potential conflict of interest.

## Footnotes

1 Davidson (1967, p. 179) writes that “events have a unique position in the framework of causal relations.”

2 A more detailed classification is presented by Lombard and Gärdenfors (2017) and Gärdenfors et al. (2018).

3 The precise definition is: A mapping *f: X→Y* between topological spaces is called *continuous* if the pre-image under *f* of any open subset of *Y* [denoted *f^–1^(Y)*] is an open subset of *X*. I should be noted that any metric induces a nearness relation.

4 Again, a metric induces a betweenness relation. If S is a space with a metric d, then *z* in *S* lies *between x, y* in *S* if *d(x,y)* = *d(x,z)* + *d(z,y).*

5 Gärdenfors et al. (2018) argue that these constraints are central for the ‘working model’ of an event (Zacks et al., 2007; Radvansky and Zacks, 2014).

6 Pearl’s (2000) model requires that the causal structure of the variables is provided in advance.
